# Phase II study of autologous mRNA electroporated dendritic cells (TriMixDC-MEL) in combination with Ipilimumab in patients with pretreated advanced melanoma

**DOI:** 10.1186/2051-1426-2-S3-P129

**Published:** 2014-11-06

**Authors:** Kris Thielemans, Bart Neyns, Sofie Wilgenhof, Jurgen Corthals, Carlo Heirman

**Affiliations:** 1 Medical School Vrije Universiteit Brussel (VUB), Brussels, Belgium; 2 University Hospital VUB, Brussels, Belgium

## Background

Autologous monocyte-derived mRNA electroporated dendritic cells (TriMixDC-MEL) are immunogenic and have anti-tumor activity in patients (pts) with pretreated advanced melanoma (Wilgenhof S. et al. Ann Oncol. 2013). Ipilimumab (Ipi) is a monoclonal antibody directed against the CTLA-4 receptor that counteracts physiologic suppression of T cell function and improves the overall survival of pts with advanced melanoma.

## Methods

The activity and safety of TriMixDC-MEL (4.106 cells id and 20.106 iv, q3wks x4) combined with Ipi (10 mg/kg q3wks x4), followed by Ipi maintenance therapy (10 mg/kg q12w, in pts who are progression-free at week 24) was investigated in pts with advanced pretreated melanoma according to a Simon two-stage Phase II study design. The primary endpoint was the 6-mths disease control rate (DCR) by irRC.

## Results

39 pts initiated study treatment (16F/23M; median age 46y [range 24-70]; AJCC stage IIIc/IV M1a/-M1b/-M1c: 1/6/4/28; prior therapy: BRAF- or MEK-inhibitor: 23 pts, chemotherapy: 18 pts). Following DC-administration, gr2 skin injection site reactions were observed in all pts, post-infusion chills (< gr2) in 15 (38%), and transient flu-like symptoms (< gr2) in 33 pts (85%). Most frequent grade 3/4 adverse events of special interest were: dermatitis (2 pts [5%]); diarrhea/colitis (2 pts [5%]), hypophysitis/hypopituitarism (7 pts [18%]), hepatitis (5 pts [13%]), and pneumonitis (3 pts [8%]). Systemic corticotherapy was used to treat irAE in 18 pts (46%). Best overall tumor response by irRC: 8 CR, and 7 PR (BORR 38%), 6 SD and 18 PD. All CR, and 3 PR are ongoing after a median duration of 19 mths (range 3-29 mths). The 6-mths DCR by irRC is 50% (95% CI 34-66). Median PFS and OS are respectively 6,2 (95% CI 3-9), and 14,4 mths (95%CI 10-18).

## Conclusion

This Phase II trial of autologous mRNA electroporated dendritic cells in combination with Ipilimumab in patients with pretreated advanced melanoma achieved its primary endpoint and resulted in an encouraging rate of durable tumor responses. Further clinical investigation of autologous mRNA electroporated DC-therapy in combination with immune checkpoint modulators is warranted.

